# Between now and later: a mixed methods study of HPV vaccination delay among Chinese caregivers in urban Chengdu, China

**DOI:** 10.1186/s12889-024-17697-6

**Published:** 2024-01-15

**Authors:** Vivian Wan-Cheong Yim, Qianyun Wang, Yifan Li, Chuanyun Qin, Weiming Tang, Shenglan Tang, Mark Jit, Jennifer S. Smith, Heidi J. Larson, Joseph D. Tucker, Jing Li, Leesa Lin, Dan Wu

**Affiliations:** 1 Department of Obstetrics & Gynaecology, Tuen Mun Hospital, Hong Kong, China; 2 Department of Social Welfare, University of California, Los Angeles, USA; 3 West China School of Public Health and West China Fourth Hospital, Sichuan University, Chengdu, China; 4 School of Medicine, University of North Carolina at Chapel Hill, Chapel Hill, NC USA; 5 SESH (Social Entrepreneurship to Spur Health) Team, Guangzhou, China; 6 Global Health Research Centre, Duke Kunshan University, Jiangsu, China; 7 Duke Global Health Institute, Duke University, Durham, NC USA; 8 Centre for Mathematical Modelling of Infectious Diseases, London School of Hygiene & Tropical Medicine, London, GB UK; 9 Department of Epidemiology, UNC Gillings School of Global Public Health, Chapel Hill, NC USA; 10 Department of Infectious Disease Epidemiology, London School of Hygiene & Tropical Medicine, London, GB UK; 11 Institute for Health Metrics and Evaluation, University of Washington, Seattle, USA; 12 Faculty of Infectious and Tropical Diseases, London School of Hygiene & Tropical Medicine, Room 360, Keppel St, London, WC1E 7HT UK; 13 Laboratory of Data Discovery for Health Limited (D24H), Hong Kong Science Park, Hong Kong SAR, China; 14 WHO Collaborating Centre for Infectious Disease Epidemiology and Control, School of Public Health, LKS Faculty of Medicine, The University of Hong Kong, Hong Kong SAR, China; 15 Department of Social Medicine and Health Education, School of Public Health of Nanjing Medical University, No. 101 Longmian Avenue, Nanjing Jiangsu, China

**Keywords:** Cervical cancer, Human papillomavirus, Vaccine delay, China, Mixed methods

## Abstract

**Background:**

Adolescent girls in China have a low HPV vaccination rate. Although vaccination is recommended by the Chinese health authorities, the cost is not covered by the national immunisation programme. Vaccination delay, among other reasons such as supply shortage and poor affordability, may contribute to low uptake. This sequential mixed methods study aimed to identify potential factors of delayed HPV vaccination among Chinese adolescent girls.

**Methods:**

Quantitative data about the attitudes and perceptions of HPV vaccination were collected from 100 caregivers of 14–18-year-old girls using an online survey in Chengdu, China. The survey data informed a subsequent qualitative study using four focus group discussions. We conducted a descriptive analysis of the survey data and a thematic analysis of the qualitative data. The findings were interpreted using a health behaviour model adapted from the Health Belief Model and the Andersen’s Behavioural Model for Health Services Use.

**Results:**

A total of 100 caregivers – 85 were mothers and 15 were fathers – participated in the survey; 21 caregivers joined focus group discussions. When asked about their intended course of action if the 9vHPV vaccine was out-of-stock, 74% chose to delay until the 9vHPV vaccine is available while 26% would consider 2vHPV or 4vHPV vaccines or seek alternative ways to procure the vaccine. Qualitative results confirmed that caregivers preferred delaying HPV vaccination for adolescent girls. The intent to delay was influenced by systemic barriers such as supply shortage and individual-level factors such as a preference for the 9vHPV vaccine, safety concerns, inadequate health communication, and the belief that adolescents were unlikely to be sexually active.

**Conclusion:**

In urban areas, Chinese caregivers’ intent to delay vaccination in favour of 9vHPV vaccine over receiving the more accessible options was influenced by a mix of individual and contextual factors. Focussed health communication strategies are needed to accelerate HPV vaccination among adolescents.

**Supplementary Information:**

The online version contains supplementary material available at 10.1186/s12889-024-17697-6.

## Introduction

In 2020, China accounted for approximately 18% of the global burden of new cervical cancer cases and over 15% of attributable deaths due to the disease worldwide [1, 2]. With over 170 women dying from cervical malignancy daily [1, 2], the disease poses a significant threat to women’s health in China. One of the safest and most effective tools for primary prevention of cervical cancer is the HPV vaccine [3]. However the current HPV vaccination rate for 9–14 year old girls, the target population for primary prevention according to WHO guidelines, is less than 5% in China [4] and far behind the WHO 2030 target of achieving full vaccination among 90% of eligible girls by 15 years of age [3]. In 2019, only 11% of young female college students aged 16 and above self-reported having been vaccinated against HPV with lowest coverage in Western China at 8.6% [5]. Currently, there are five vaccines available in the Chinese market: there are four options for eligible females 9–45 years old including a domestically produced bivalent vaccine (Cecolin®) and three imported vaccines (bivalent 2vHPV, quadrivalent 4vHPV and 9-valent 9vHPV), and for females aged 9–30 years there is another domestic bivalent option (Walrinax®) [5, 6]. Adolescent girls between 9–14 years old require two shots to complete the vaccine series, while those beyond this age group require three shots [7]. Bivalent vaccines target HPV16 and 18, which are responsible for 84.5% of cervical cancer in China [8]. The 9vHPV vaccine targets five additional high-risk HPV subtypes; together with HPV16 and 18 they cause approximately 90% of cervical cancers [9].

Major barriers to HPV vaccination in China include high cost of vaccines [2, 10, 11] and supply shortage of imported vaccines compared to domestic options [10–12]. Since the HPV vaccine is not included in the National Immunisation Programme [10], consumers largely pay out-of-pocket and can choose which product to receive [6]. The 9vHPV vaccine costs 190.3 USD per dose, while the domestic bivalent vaccine only costs 48.2 USD per dose [2, 13]. Despite the greater availability and lower price of domestic 2vHPV vaccines, Chinese caregivers preferred 9vHPV vaccines [12]. The reasons for this preference were not well studied. Existing literature suggest many perceived higher valent vaccines to be more effective in cervical cancer prevention, a belief further reinforced by media coverage of their unavailability [4, 12]. Desperation and persistent shortages led to smuggling, informal markets and vaccine tourism which limited accessibility for those who needed it the most [12, 14, 15]. Others opted to delay vaccination well past the recommendation of 9–14 years to obtain higher valent vaccines, thus were at risk of compromising the vaccine’s protective benefits, which worked best before HPV infection was acquired. For the purposes of this study, vaccine delay is defined as the expressed intention to delay initiation of the HPV vaccine series until a future, unspecified time in favour of 9vHPV vaccines despite lower valent products being available.

Vaccine delay can be understood as a form of vaccine hesitancy, a psychological state of uncertainity regarding vaccination that is affected by multiple personal and macro-level factors linked to population health literacy, socio-cultural beliefs, and vaccine and health-related policies and programmes independent of behaviour [16–18]. For Chinese adolescents awaiting HPV vaccination, primary caregivers, usually parents, have a strong influence over the decision [19–22]. In 2020, Zheng et al. found that more than half of guardians of secondary school girls in China were vaccine-hesitant; among them 21.1% reported they were undecided about vaccination [23]. Studies found that Chinese caregivers were primarily influenced by concerns about vaccine safety and effectiveness [6, 16, 24], inadequate access to health information and professional advice [16, 24] and low risk perception of cervical cancer [6]. To increase early uptake of available HPV vaccines among adolescent girls, caregivers’ concerns must be addressed. Notably, not much is known about caregivers’ HPV vaccine delay for adolescents and how caregivers who chose to delay differed from those who didn’t [25], especially in the Chinese context.

This sequential mixed methods study aims to address the gap in literature by exploring the underlying individual and contextual factors behind Chinese caregivers’ decision to delay HPV immunisation using behavioural health theories. We hypothesize that vaccine delay is due to a combination of individual and contextual factors, including problems with health communication and messaging.

## Methods

### Study design

An explanatory sequential mixed methods design was used to connect the quantitative and qualitative phases of the study by selecting participants for focus group discussions and through development of interview guides grounded in the results of the quantitative phase [26]. Quantitative data about caregivers’ attitudes and perceptions of HPV vaccination were initially collected using surveys. Subsequent analysis of quantitative data revealed a general overview of caregivers’ attitude, specifically a preference for 9vHPV vaccines. The qualitative phase was designed to provide a richer interpretation of caregivers’ perspectives, which cannot be adequately explained by reporting quantitative results alone. The sampling strategy and topic guide for focus groups were built on quantitative findings, and aimed to study the effects of individual and contextual factors on the attitude towards HPV vaccination using a health behaviour framework and thematic analysis of qualitative data [26].

### Study site and population

The study was conducted in Chengdu, the capital city of Sichuan Province in Western China with a population of over 21 million [27]. Previous studies conducted in China observed regional differences in incidence rate of cervical cancer and HPV vaccination uptake according to socio-economic status [5]. Urbanized, developed coastal regions in Eastern China had higher HPV vaccination coverage compared to poorer Western regions, which had the lowest coverage among adolescent girls 16 years and above [5]. The study population consisted of primary caregivers of 14–18-year-old adolescent girls living in the Wuhou district of Chengdu, one of the most developed areas in China. We focussed on this age group because they were part of an important catch-up population that had not been included in the on-going government subsidized HPV vaccination programmes [28]. Inclusion criteria were self-identified primary caregivers (including parents, guardians, or anyone primarily responsible for childcare) of adolescent girls (aged 14–18 years) who were clinically eligible for the vaccine and living in Chengdu at the time of study. Primary caregivers of adolescent girls below 14 or above 18 years old, whose girls were ineligible to receive the vaccine based on clinical evaluation, and not living in Chengdu at the time of study were excluded.

### Quantitative phase

We collected quantitative data from 100 caregivers about their perceptions and practice concerning HPV vaccination from January 4th to February 18th, 2022 as part of a pre-intervention survey of a pilot study aiming to test the feasibility of an intervention to increase HPV vaccination among Chinese girls [29]. Specifically, baseline socio-demographic data and information about attitude towards HPV vaccination were collected. Survey items were developed based on previous vaccine-related literature [30] and adapted to focus on HPV vaccination (Additional file 1: Appendix S1 Survey items). A local community health centre in Chengdu distributed our pilot information online and interested individuals voluntarily visited the centre to participate in the pilot. Caregivers of eligible participants were invited to complete an online survey lasting approximately 15 min at the health centre before participating in the pilot. Analysis of the survey data is included in this manuscript (Additional file 2: Appendix S2 A descriptive summary of the variables from the baseline survey). Preliminary findings noted a preference for the 9vHPV vaccine among caregivers, and generated questions about 1) caregivers’ perception of the 9vHPV vaccine compared to other products, 2) their understanding of the benefits and barriers of vaccination, 3) how HPV vaccine-related information were communicated and caregivers’ interpretation of such messages, and 4) the effect of contextual factors on their attitude towards immunization. To explore these questions in greater depth, the qualitative phase was developed.

### Qualitative phase

Four focus group discussions lasting between 60–90 min were conducted between June 8th to August 12th, 2022 (Additional file 3: Appendix S3 Topic guide for focus group discussions). A total of 21 caregivers were recruited. We circulated an invitation for focus group discussion via a local social medial platform among caregivers of adolescent girls who had commenced the HPV vaccine series under the pilot study. We used purposive sampling to recruit a sub-set of caregivers (*n* = 11) from the pilot. To capture the views of caregivers of adolescent girls who had yet to be inoculated, we recruited a further ten participants via snowball sampling (*n* = 10) using the same invitiation via the same social media platform. The same inclusion/exclusion criteria described above were used for recruitment. Male participation was encouraged by asking female participants to involve their partners and spread the recruitment call among their peer groups. Interested individuals would then be contacted by a member of the research team with experience in community engagement and qualitative research. The first three focus groups were conducted face-to-face, but the final focus group was shifted to an online, audio format due to safety concerns over the COVID-19 pandemic. All focus group discussions were facilitated in Mandarin by two moderators with experience in qualitative research, and audio recorded for purposes of data analysis. Written informed consent was obtained for all in-person activities and substituted with verbal consent when online. Each participant was offered a remuneration of 150 RMB (21.4USD) after the completion of discussions.

### Theoretical framework

Available literature about HPV vaccine uptake and health behaviour theories were used to guide the focus group design and interpret vaccination behaviour. The Health Belief Model (HBM) is a popular framework used in vaccination studies [31, 32], but it mainly focuses on the six constructs affecting an individual’s assessment of a health risk and the benefits and barriers to vaccination [33, 34]. We needed a model that gave weight to both individual and contextual determinants, like socio-cultural, structural, and systemic elements (i.e., society’s attitude towards sex, population health literacy, vaccine supply). The Andersen’s Behavioural Model of Health Services Use (BMHSU) considers both aspects to understand how people come to use a health service, and has been used to examine how caregivers interact with health systems and providers when deciding to vaccinate their daughters against HPV [35].

**Fig. 1:**
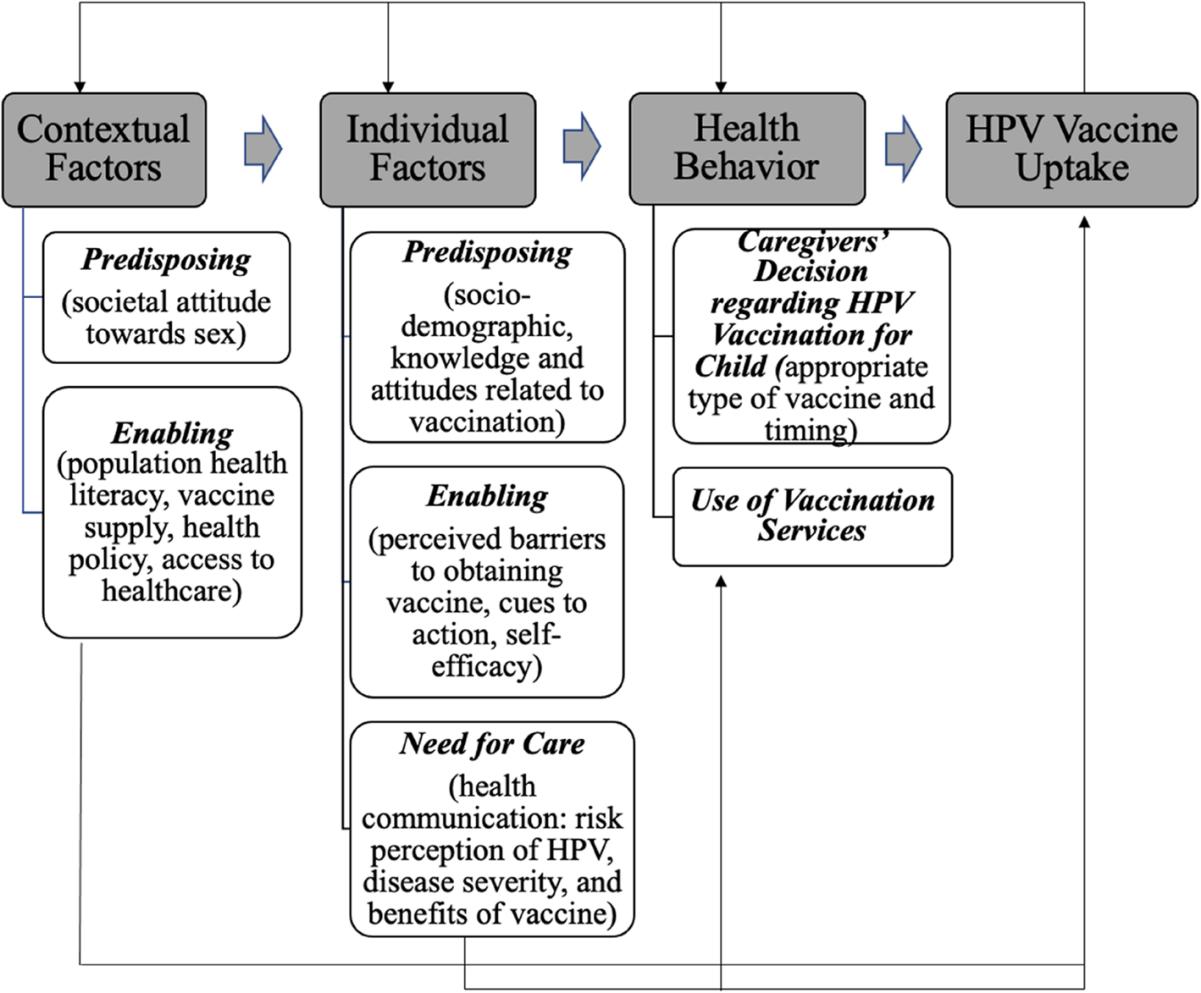
An Integrated Model to Examine Caregivers’ Decision to Vaccinate their Daughter [33, 36]

We adapted components of the Health Belief Model into the Anderson’s model to guide the design of focus groups and interpretation of findings (Fig. 1). The six constructs of the Health Belief Model framed the understanding of individual factors. The predisposing, enabling and need for care domains of the Anderson’s were retained [36]. Predisposing factors affecting the individual included socio-demographic factors (i.e., gender, education), while predisposing contextual factors involved cultural norms and attitudes (i.e., adolescent sexuality) [37]. Enabling factors affected both individual (i.e., perceived barriers, cues to action) and contextual conditions (i.e., vaccine supply, health policies), and were defined as any logistical factors that affected HPV vaccination [37]. Finally, the need for care domain looked at how caregivers assessed the child’s susceptibility to HPV and cervical cancer, the severity of consequences if infected and the benefits of the vaccine against its risks [37].

### Analysis

We focussed on understanding how caregivers who chose to delay differed from those who did not. Caregivers were divided into “delay” and “no delay” groups based on their response to a question in the baseline survey prior to participating in the pilot intervention. Caregivers were asked how they would act given the hypothetical scenario in which the 9vHPV vaccine was out of stock in their community health centre. The “delay” group included those who answered they would wait for availability of 9vHPV and those who gave no response because inaction or no decision were interpreted as forms of delay. The “no delay” group included those who said they would choose 2vHPV, 4vHPV or use alternative methods to obtain the vaccine. We looked for any association between caregivers’ socio-demographic characteristics, perception of vaccination benefits, barriers and sources of health information and their decision to delay or not delay vaccination. Descriptive analysis was performed using STATA/SE v.17 (Statacorp, Texas, USA) using Chi-square test and Fisher’s exact test where appropriate, and a *p*-value < 0.05 was considered statistically significant.

Qualitative data were analysed using thematic analysis with a coding reliability approach [38]. The coding frame included pre-determined codes, identified through available literature [6, 24, 39] and health behaviour theories. Codes that emerged from data familiarisation were also included. Two independent coders using NVivo 12 applied the coding frame to all transcripts using an inductive and deductive approach. When there was disagreement, a third coder with extensive qualitative experience reviewed coding discrepancies until consensus was reached. We defined themes as overviews that highlighted the most prominent issues raised by participants in relation to a topic [38], and they emerged from triangulating patterns in the data between independent coders and contextualizing findings within health behaviour theories. Data saturation was reached upon finding recurrent themes with no new findings generated. Relevant Mandarin Chinese quotations were translated into English for reporting.

To ensure rigorous report of mixed methods research, we adhered to the good reporting of mixed methods study (GRAMMS) checklist throughout the design, data collection, reporting and discussion of results [40] (Additional file 4: Appendix S4 Good Reporting of A Mixed Methods Study (GRAMMS) Checklist).

## Results

### Study participants’ characteristics

In total, 100 primary caregivers completed a baseline survey for the pilot interventional study, which collected information about socio-demographic characteristics, perceived benefits, barriers, and health information sources. We observed that 87% of participants said they would consider delaying vaccination for reasons other than allergy or lack of eligibility. In fact, when asked about their intentions if the 9vHPV vaccine is out-of-stock, 74% of caregivers chose to delay until the vaccine is available while only 26% would consider 2vHPV or 4vHPV vaccines or seek alternate ways to procure the vaccine. We studied the reasons behind caregivers’ decision to delay vaccination by comparing caregivers who chose to delay with those who did not. Our results showed that both “delay” and “no delay” groups – hereby known as delayers and non-delayers – had similar baseline characteristics presented in Table 1.

**Table 1:** Comparison of caregivers’ demographic characteristics based on decision to delay or not delay vaccination

		**Caregiver’s Decision to Delay HPV Vaccination**		
**Characteristics**	**Total** ***n* = 100**	**Delay (%)** ***n* = 74**^**a**^	**No Delay (%) *n* = 26**^**b**^	***p*-value**
**Age of Primary Caregiver**				
**≤ 45 years old**	50/96 (52%)^c^	42 (57.5%)	8 (34.8%)	0.057
**> 45 years old**	46/96 (48%)^c^	31 (42.5%)	15 (65.2%)	
***Mothers* ≤ *45 years old***^**e**^	38/77 (49%)^f^	32 (57.1%)	6 (28.6%)	0.026
***Mothers* > *45 years old***^**e**^	39/77 (51%)^f^	24 (42.9%)	15 (71.4%)	
**Gender of Primary Caregiver**				
**Male**	16/100 (16%)	13 (17.6%)	3 (11.5%)	0.471
**Female**	84/100 (84%)	61 (82.4%)	23 (88.5%)	
**Marital Status**				
**Married**	90/100 (90%)	66 (89.2%)	24 (92.3%)	0.648
**Divorced, unmarried or other**	10/100 (10%)	8 (10.8%)	2 (7.7%)	
**Education Level**				
**Primary school and/or below**	3/100 (3%)	2 (2.7%)	1 (3.9%)	0.957
**Secondary school**	27/100 (27%)	20 (27.0%)	7 (26.9%)	
**University and/or above**	70/100 (70%)	52 (70.3%)	18 (69.2%)	
**Annual Household Income RMB/year (USD)**				
**0–80000 RMB (< 12,560 USD)**	42/100 (42%)	31 (41.9%)	11 (42.3%)	0.963
**80,000–300000 RMB (12,560–47096 USD)**	49/100 (49%)	36 (48.7%)	13 (50.0%)	
**More than 300,000 RMB (≥ 47,096 USD)**	9/100 (9%)	7 (9.4%)	2 (7.7%)	
**Employment Status**				
**Unemployed**	15/100 (15%)	13 (17.6%)	2 (7.7%)	0.225
**Employed**	85/100 (85%)	61 (82.4%)	24 (92.3%)	

^a^Delay group included 10 participants who did not answer the question about whether to delay vaccination if 9vHPV vaccine is out of stock in their community centre because inaction or unknown action is interpreted as delay in decision-making

^b^No delay group included everyone who opted for 2vHPV, 4vHPV and alternative methods of obtaining vaccine

^c^Four missing responses in total for age, one from “delay” group and three from “no delay” group

^e^Analysis of a subset of respondents who are mothers

^f^Three missing responses in total for age, one from “delay” group and two from “no delay” group

A total of four focus groups were conducted – twenty mothers and one father participated. Most participants had adolescent daughters between 14–18 years old (mean age 16.3 years, SD = 1.5).

### Caregivers’ attitude towards HPV vaccination delay

Concerning why caregivers decide to delay HPV vaccination for their children, three recurring inter-related themes emerged from focus group discussions summarised in Table 2: role of parents in decision-making, preference for 9vHPV vaccine and barriers to timely vaccination. Clear boundaries separating themes were not always apparent, thus highlighting the fluid, complex nature of vaccine delay. We reported these results alongside quantitative findings in Table 3, which compared the perceptions of vaccination held by delayers and non-delayers. A summary of the quotes used in the qualitative analysis and their sources is presented (Additional file 5: Appendix S5 A summary of the quotes from the qualitative analysis and their sources).

**Table 2:** Overview of factors influencing vaccination delay in urban Chengdu, China based on focus group discussions

Major Theme	Subtheme
**Role of parents**	Female dominant role in decision-making
**Preference for 9vHPV vaccine**	Perceived benefits of HPV vaccination “Once and for all” option
**Barriers to timely vaccination**	Concerns over vaccine safety Inadequate health communication Vaccine shortage
	Knowledge gap and misinformation Perceived sexual inactivity of adolescent girls

**Table 3:** Comparison of caregivers’ attitudes based on decision to delay or not delay HPV vaccination

	**Caregiver’s Decision to Delay HPV Vaccination**		
**Survey Item**	**Delay (%)**^**a**^ ***n* = 74**	**No Delay(%)**^**b**^ ***n* = 26**	***p*-value**
***Perceived Vaccination Benefits***			
** “I want to protect my child against cervical cancer”**			
Yes	63/74 (85.1%)	25/26 (96.2%)	0.137
No	11/74 (14.9%)	1/26 (3.8%)	
** “I believe HPV vaccine is important”**			
Disagree	1/74 (1.4%)	0/26 (0%)	0.380^c^
Agree	25/74 (33.8%)	13/26 (50%)	
Strongly agree	48/74 (64.9%)	13/26 (50%)	
** “I believe the vaccine is safe”**			
Agree	44/74 (59.5%)	18/26 (69.2%)	0.380
Strongly agree	30/74 (40.5%)	8/26 (30.8%)	
** “I believe the vaccine is effective”**			
Agree	42/74 (56.8%)	17/25^e^ (68%)	0.322
Strongly agree	32/74 (43.2%)	8/25^e^ (32%)	
** “There is known history of HPV infection in my family”**			
Yes	1/74 (1.4%)	2/26 (7.7%)	0.103
No	73/74 (98.7%)	24/26 (92.3%)	
** “There is known history of cervical cancer in my family”**			
Yes	1/74 (1.4%)	0/26 (0%)	1.000^c^
No	73/74 (98.7%)	26/26 (100%)	
***Perceived Vaccination Barriers***			
** “Cost of the vaccine is a barrier”**			
Yes	11/73^e^ (15.1%)	5/26 (19.2%)	0.621
No	62/73^e^ (84.9%)	21/26 (80.8%)	
** “I have heard of negative news related to HPV vaccines in the media”**			
Yes	12/73^e^ (16.4%)	4/26 (15.4%)	0.900
No	61/73^e^ (83.6%)	22/26 (84.6%)	
** “I have friends and/or family who oppose to getting the HPV vaccine”**			
Yes	8/73^e^ (11%)	4/26 (15.4%)	0.553
No	65/73^e^ (89%)	22/26 (84.6%)	
** “People in my social circle have had bad experience with the HPV vaccine**			
Yes	3/73^e^ (4.1%)	0/26 (0%)	0.564^c^
No	70/73^e^ (95.9%)	26/26 (100%)	
***Sources of Health Information***			
** “I have been given recommendations from friends and/or family”**			
Yes	19/74 (25.7%)	10/26 (38.5%)	0.216
No	55/74 (74.3%)	16/26 (61.5%)	
** “I have been given recommendations from healthcare worker(s)”**			
Yes	17/74 (77%)	9/26 (34.6%)	0.244
No	57/74 (23%)	17/26 (65.4%)	

^a^Delay group included 10 participants who did not answer the question about whether to delay vaccination if 9vHPV vaccine is out of stock in their community centre because inaction or unknown action is interpreted as delay in decision-making

^b^No delay group included everyone who opted for 2vHPV, 4vHPV and alternative methods of obtaining vaccine

^c^Fisher’s exact test

^e^ One missing response

### Role of parents

Our results noted parents had a significant role to play in the vaccination decision. Indeed, 94% (94/100) of primary caregivers participating in the survey were parents, with mothers making up 85% (80/94) and fathers 15% (14/94) of the parent population. Triangulation of quantitative and qualitative findings found that mothers were responsible for nearly the entire decision-making process when it came to deciding how, when and if their daughters got vaccinated. Further analysis based on maternal age found more young mothers (at/below 45 years old) among the delayers (57.1%) compared to the non-delayers (28.6%) (*p*-value = 0.026). When asked what role their male partners played, most mothers said their partners were not actively involved and mainly played a “supporting role”. One mother said, “[my husband] didn’t even know the vaccine existed – never heard of it before – there was no need for fathers to pay attention.” Indeed, one father admitted he only found out about the vaccine through our recruitment call and was unfamiliar with the types of available vaccines in the market, the differences in benefit and effectiveness.

### Preference for the 9vHPV vaccine

Survey findings revealed that most parents believed the HPV vaccine to be safe, effective, and important regardless of whether they chose to delay vaccination or not. However, focus group discussions observed that when asked how caregivers assessed one HPV vaccine was better than another, most associated higher coverage of HPV subtypes with much greater protection against cervical cancer. The 9vHPV vaccine was seen as the “once and for all” option compared to bivalent and quadrivalent vaccines among caregivers because it was “the best option out there”. During focus group discussions, one mother said, “rather than fighting off two or four enemies, the 9vHPV vaccine is capable of fighting nine enemies in one go.” Most caregivers said they aimed to vaccinate their daughters with the 9vHPV vaccine because it was seen as superior to 2vHPV and 4vHPV vaccines. One mother said the 2vHPV vaccine was “the most basic of options because it only covered two subtypes of HPV viruses”. Another acknowledged the immense difficulty of obtaining the shot, and said she was resigned to “make do with a lesser option, like the bivalent vaccine” for her daughter if the preferred 9vHPV vaccine remained out-of-reach. This opinion of the 9vHPV vaccine being superior to its counterparts was linked to the perception that the higher the coverage the better the vaccine, and secondly, lower coverage vaccines were relegated to substitutes when 9vHPV vaccines were unavailable. Despite the higher cost of 9vHPV vaccines caregivers saw it as the best option available in the market, and said they were “undeterred by the cost because it was for the good of [their] child”.

### Barriers to timely vaccination

#### Inadequate health communication

When asked how caregivers initially became aware of the HPV vaccine, the most common information source was word of mouth from others within their social circle. Our survey found that more non-delayers (38.5%) reported having received recommendations from friends and/or family regarding vaccination compared to delayers (25.7%). Focus group discussions revealed this was often supplemented by other channels of communication like *baidu* (internet search engine), a plethora of individual and public social media accounts (Tik Tok, Weibo, WeChat public pages etc.) and Chinese news media outlets all competing for caregivers’ attention. Although survey findings suggested most participants reported no opposition from friends/family towards HPV vaccination, few people from their social circle having had an adverse vaccination experience and most denied hearing negative rumours in the media, the process of obtaining information to make an informed decision was described as “overwhelming”, “messy” and “confusing”. One parent described the frustration,

“Most of the information is passed on by word of mouth because it is a hot topic among our friends. It’s hard to trust all the information – we must take everything that was said with a grain of salt.” *(Mother of vaccinated daughter, focus group 1)*

The opinion of getting 9vHPV vaccine was popular among parent circles. One mother said this was “exacerbated by extensive media coverage of the massive shortage, which led to the perception it must be the best option available”. Another mother said, “When I attended the appointment, I had no idea what to choose. I was just going to get whatever other people got because I had no idea.” Healthcare providers struggled to bridge the health information gap. Although many caregivers said they preferred to obtain information about HPV and related vaccination directly from “sources of authority” like local health authorities and healthcare providers, most admitted there were no opportunities to discuss the benefits of vaccination, how to choose between different vaccines and optimal timing of vaccination with healthcare providers.

#### Supply shortage

Most of the caregivers cited the lack of 9vHPV vaccine supply as an important barrier to vaccination. For each vaccination site, there were tens of thousands of people fighting for one of the two to three thousand 9vHPV shots annually. To obtain 9vHPV vaccination appointments, caregivers needed to pay attention to daily release of vaccination quotas like *miaosha* (i.e., booking a vaccine within seconds) via government approved online platforms, with success boiling down to a combination of speed and luck. Several caregivers described setting regular alarms for months to remind themselves to go online, while others waited for years, with little success. One mother said,

“Everyone, from the government to the health professionals, all said the 9vHPV vaccine was the best. We were told to get vaccinated as early as possible, but this was impossible because there were no vaccines available!” (*Mother of vaccinated daughter, focus group 1)*

Some caregivers reported knowing someone who took drastic action by travelling to Hong Kong to get the vaccine, while others reported hearing about illegal activities such as scalping – the resale of 9vHPV vaccines at exorbitant prices – and fraud.

#### Perceived sexual inactivity of adolescent girls

Most caregivers acknowledged it was best for children to vaccinate before sexual debut and had heard the suggestion to vaccinate “the earlier the better”. However, some caregivers thought it was more important to vaccinate with the preferred product. Nearly all caregivers cited senior secondary school – equivalent to 16 to 18 years old – as an appropriate age range to receive the vaccine and were confident their children were unlikely to be sexually active up to that stage. Several mothers were aware that children could receive the vaccine starting at the age of nine, but thought the recommended age was too early because there is no urgency to vaccinate straight away. One mother said, “before the age of 18, children are at school or at home so there won’t be any major problems as they’re under our noses all the time.” Most caregivers favoured university as the ideal cut-off. One parent said,

“Sixteen years old is an ideal age to get vaccinated because we can get this done before she goes off to university. After kids leave for university, no matter how strict you are with them before, everything is out of your control. I cannot guarantee she will not engage in sexual activity then, can I?” *(Mother of vaccinated daughter, focus group 1)*

#### Knowledge gaps and misinformation

When asked about the benefits of the vaccine, the most consistent piece of knowledge accurately identified by caregivers was that it reduced the risk of cervical cancer. Beyond that, only one person could identify protection against genital warts and very few knew it protected males against infection. Very few caregivers understood different subtypes of HPV led to different health consequences. Only one person recognized all available vaccines protected against high-risk subtypes, was able to name the two most common cancer-causing subtypes, and acknowledged the 2vHPV vaccine is highly effective against them. Additionally, some caregivers incorrectly said the vaccine can “protect against HIV/AIDS”, and “reduce the risk of breast cancer”.

#### Concerns about vaccine safety

The reliability of vaccine manufacturer was a significant safety concern. Even though opportunities to obtain the elusive 9vHPV vaccine through private social media platforms, private hospitals, medical tourism, and scalping existed, caregivers said “they did not dare use them” because the source of vaccine was “highly questionable”. The majority would only trust vaccines that came from community health centres, government hospitals, or media platforms associated with local health authorities. When asked whether caregivers considered whether a vaccine was domestically produced or imported, the consensus was that people leaned more towards imported vaccines because the 9vHPV and 4vHPV vaccines were only produced abroad. Whether a genuine preference for imported vaccines existed was unclear. As for vaccine effectiveness, some caregivers were able to accurately identify the 9vHPV vaccine prevented more than 90% of HPV-attributable cervical cancer while other vaccines protected against 70–80%. Few expressed significant doubts about HPV vaccine effectiveness, and most found it acceptable.

## Discussion

This study highlights that Chinese caregivers’ plan to delay their daughters’ HPV vaccination over receiving the more accessible options was influenced by a mix of individual and contextual factors. Our integrated health behaviour model offers a novel way of visualizing how these factors are interrelated. We found that perceived sexual inactivity of adolescents [35, 41–43], insufficient knowledge about vaccination timing, safety and effectiveness [25, 43–45] and preference for 9vHPV vaccine influenced parents’ individual decision and were consistent with determinants of vaccine hesitancy identified from studies conducted in the United States, Kenya and Japan. Furthermore, supply shortage, inadequate communication and dissemination of information [42–44] also contributed to hesitancy. Our findings suggest that vaccine hesitancy may encourage delayed vaccination among Chinese caregivers. WHO SAGE recommended use of focussed health communication strategies and messaging to address hesitancy and improve vaccine uptake [46], and this paper presented the following recommendations for consideration.

Our data suggest a Chinese caregiver preference for 9vHPV vaccines, which is consistent with data from China [21, 47]. This is related to the perception that higher coverage of HPV viruses offered much greater effectiveness [48]. The key is to strike a balance between vaccinating with lower-valent but generally available vaccine types at a younger age, versus waiting for higher-valent vaccines to become available with the risk jeopardizing protection due to delayed vaccination. Recent evidence found that protection against cervical cancer declined as the initial age of receiving the vaccine increased [49], meaning that a delay in initiating vaccination can compromise overall protection effects against cervical cancer. Our findings also suggest a lack of awareness of the implications of different HPV subtypes, the health consequences of high-risk oncogenic types versus low-risk types among caregivers, which affected their ability to fully grasp the additional benefits conferred by a higher-valent vaccine, such as protection against genital warts. To address this issue, public health messages targeted towards caregivers of adolescent girls, especially parents, should avoid overemphasis on the numerical difference in coverage (i.e., number of HPV viruses covered), clearly communicate all vaccine types are effective against high-risk HPV and emphasize available vaccine products in the market (i.e., 2vHPV vaccine) as equally acceptable. This can be combined with the ongoing efforts from Chinese health authorities and experts calling for greater importance to be placed on timely vaccination at an appropriate age rather than waiting for available 9vHPV vaccines [7].

Our findings also suggest that caregivers believed timely vaccination at the recommended age was unnecessary because they believed their child was too young to be sexually active nor would likely be sexually active soon. Similar findings were found in studies conducted in the United States [34, 39]. Due to its sexually related mode of transmission, effective communication around the HPV vaccine needed to be culturally appropriate for messages to be well received in Chinese society, which holds a conservative attitude towards sex. Our data suggest caregivers lacked awareness of the scientific rationale behind timely vaccination and questioned the safety of vaccinating at a young age. Notably, there are few high quality research evaluating the effectiveness of communication interventions on HPV vaccine uptake [50]. Some countries noted greater success referring to the vaccine as a “cancer vaccine” rather than a vaccine against a sexually transmitted disease [42]. Social networks could be leveraged to increase the reach and impact of accurate messages. Our study found that caregivers who chose not to delay vaccination were more likely to have communicated with friends or family members. This is supported by studies in Japan showing greater intention to vaccinate among parents who had the opportunity to discuss the HPV vaccine with their peers [44, 45]. Parents who thought their friends had a positive attitude towards the vaccine were more motivated to inoculate their children [44, 45]. Our data suggested that Chinese caregivers were most likely to be introduced to the vaccine by their peers. Given this finding, it may be worthwhile for future studies to investigate the effectiveness of different methods for dissemination of health information among parents, specifically how to leverage social support networks to dispel misinformation and promote the message of timely vaccination.

Currently, there is a lack of evidence-based communication strategies around HPV vaccination, and more research is needed to bridge this knowledge gap. Although the internet and social media were popular ways to obtain information, their roles in vaccine decision-making were not fully understood [46]. A study looking at Chinese social media portrayal of available HPV vaccines found that some descriptions of the vaccines were inconsistent and failed to cover key epidemiological information such as high-risk HPV and non-cervical complications [12]. Additionally, the media fixation on price, vaccine shortage and difficulty of booking appointments heightened anxiety, making it difficult for the more rational advice of health professionals to be heard [12]. School-based vaccination programmes however were viewed favourably because they inspired confidence, increased convenience, improved HPV knowledge and the willingness to vaccinate among adolescents [51–54]. Incorporating HPV-related topics into the sexual health curriculum, in parallel with school-based vaccination programmes, may reinforce positive and accurate information about HPV vaccination.

This study had several limitations. The length of vaccination delay to vaccination was not specified and participants were not followed up to confirm completion of vaccination after initial period of delay. The quantitative part of this study was underpowered although it was not designed to provide evidence for non-inferiority. The use of convenient sampling and a small sample size meant that participants’ socio-demographic and economic backgrounds were quite homogenous. Results primarily reflected the opinions of caregivers with children vaccinated against HPV or awaiting immunisation but not those who rejected the vaccination. There is an issue of generalisability, and caution is needed when interpreting the findings in the context of the general Chinese population because this study was carried out in one of the most developed districts in Chengdu. Nevertheless, this study adds to the growing literature about HPV vaccination delay, which is limited in the Chinese context. Replicating this research using a larger, nationally representative sample and increasing male participation could be beneficial.

## Conclusion

This study underscored the complexity of caregivers’ intent to delay adolescent HPV vaccination, which came from considering multiple, interrelated individual and contextual factors. It presented another perspective to understand vaccination delay, which is a significant barrier to increasing uptake among adolescents in China. To address the issue, public health messages need to communicate the importance of timely vaccination and that all vaccine types confer protection against the most common high-risk HPV types. Further, messages need to be framed in a culturally sensitive manner and delivered through appropriate channels to adolescents and their caregivers to maximize their impact and reach. Subsequent research should focus on evaluating the effect of targeted communication strategies on adolescent vaccine uptake within different population subgroups and geographical contexts in China.

### Supplementary Information

**Additional file 1: Appendix S1. **Survey items.

**Additional file 2: Appendix S2.** A descriptive summary of the variables from the baseline survey.

**Additional file 3: Appendix S3.** Topic guide for focus group discussions.

**Additional file 4: Appendix S4.** Good Reporting of A Mixed Methods Study (GRAMMS) Checklist [40].

**Additional file 5: Appendix S5.** A summary of the quotes from the qualitative analysis and their sources.

## Data Availability

The datasets generated and/or analysed during the current study are not publicly available to protect confidentiality of participants but are available from the corresponding author on reasonable request.

## Abbreviations

HPV: Human papillomavirus
2vHPV: Bivalent vaccine
4vHPV: Quadrivalent vaccine
9vHPV: 9-Valent vaccine
HBM: Health Belief Model
BMHSU: Andersen’s Behavioural Model of Health Services Use
WHO: World Health Organization
