# Supplementary material for: Between now and later: a mixed methods study of HPV vaccination delay among Chinese caregivers in urban Chengdu, China
Source: BMC Public Health. 2024 Jan 15;24:183. doi: 10.1186/s12889-024-17697-6 (PMC10790461; doi:10.1186/s12889-024-17697-6)
Supplement: Supplementary file 1 — Additional file 1: Appendix S1. Survey items. [file 12889_2024_17697_MOESM1_ESM.docx]

**Appendix S1:** Survey items

**Part A. Basic Information**

1. What is your gender? A = male, B = female
2. What is your ethnicity? A = Han Chinese, B = Others, please state ______
3. What is your date of birth? _____________
4. What is marital status?

A = unmarried, B = married, C = divorced, D = widowed, E = others

1. What is your relationship with the participant?

A = father, B = mother, C = grandfather, D = grandmother, E = others

1. What is your education level?

A = primary school or below, B = primary school, C = secondary school, D = tertiary education, E = tertiary education and above

1. What is your occupation?

A = civil servant, B = farmer, C = blue-collar worker, D = white-collar worker,

E = technician, F = unemployed or retired, G = others

1. What is your annual familial income?

A = 0-10000 RMB/year, B = 10000-30000 RMB/year, C = 30000-80000 RMB/year，D = 80000-150000 RMB/year, E = 150000-300000 RMB/year，F = 300000-1000000 RMB/year, G = 1000000 and above RMB/year

1. Do you know anyone in your family who has been infected by HPV?

A = yes, B = no, C = I don’t know

1. Do you know anyone in your family with cervical cancer?

A = yes, B = no, C = I don’t know

**Part B. Attitude towards Vaccination**

1. The researcher gave you information about the HPV vaccine and the “pay-it-forward” program, are you willing to let your child participate in the “pay-it-forward” program?

A = yes, B = no (skip to question 13)

1. What are your reasons for agreeing to vaccinate your child? (multiple choices allowed)

A = due availability of vaccination program, B = to protect my child against cervical cancer, C = due to recommendations from friends/family, D = due to recommendations from healthcare professionals, E = due to the “pay-it-forward” program

1. What are your reasons for refusing vaccination? (multiple choices allowed)

A = insufficient knowledge about cervical cancer and HPV vaccine, B = insufficient information about HPV vaccine effectiveness, C = disapproval from family, D = unnecessary, E = worried about side effects, F = affordability, G = others, please state ______

1. Are you willing to donate money to support other girls in the community to receive the HPV vaccine? A = yes, B = no
2. How much are you willing to donate to support vaccination of other girls? (if you replied “no” to question 14, please skip this question)

A = 700 RMB (to help the next girl receive two doses of domestic 2vHPV vaccines),

B = 350 RMB (to help the next girl receive one dose of domestic 2vHPV vaccine),

C = 175 RMB

D = other amount, please state __________

1. What are the benefits of the “pay-it-forward” program? A = decrease financial burden, B = increase my understanding of the effectiveness of HPV vaccines, C = allow more girls to receive the HPV vaccine, D = decrease the spread of HPV infections, E = increase community solidarity, F = others, please state

**Part C. Attitude towards Vaccine**

1. Before this program, have you heard of the HPV vaccine? A = yes, B = no
2. I believe the HPV vaccine is important.

A = strongly disagree, B = disagree, C = agree, D = strongly agree

1. I believe the HPV vaccine is safe.

A = strongly disagree, B = disagree, C = agree, D = strongly agree

1. I believe the HPV vaccine is effective.

A = strongly disagree, B = disagree, C = agree, D = strongly agree

1. People from my social circle have received the HPV vaccine before. A = yes, B = no
2. I hesitate to receive the HPV vaccine for reasons other than allergy or sickness. A = yes, B = no
3. I would delay receiving the HPV vaccine for reasons other than allergy or sickness. A = yes, B = no
4. I would refuse the HPV vaccine for reasons other than allergy or sickness. A = yes, B = no
5. Have you heard of negative information and news about the HPV vaccine? A = yes, B = no
6. I know people within my social circle who refuse the HPV vaccine. A = yes, B = no
7. Among the people you know who have previously received the HPV vaccine, there were people who suffered from adverse effects after the vaccination. A = yes, B = no
8. The vaccine price is a barrier to vaccination for my peers and me. A = yes, B = no
9. What would you do if the community healthcare centre in your locality ran out of 9vHPV vaccines?

A = receive the 4vHPV vaccine, B = receive the domestic 2vHPV vaccine, C = receive the imported 2vHPV vaccine, D = delay vaccination until 9vHPV supply resumes, E = use other methods
