# Supplementary material for: Between now and later: a mixed methods study of HPV vaccination delay among Chinese caregivers in urban Chengdu, China
Source: BMC Public Health. 2024 Jan 15;24:183. doi: 10.1186/s12889-024-17697-6 (PMC10790461; doi:10.1186/s12889-024-17697-6)
Supplement: Supplementary file 2 — Additional file 2: Appendix S2. A descriptive summary of the variables from the baseline survey. [file 12889_2024_17697_MOESM2_ESM.docx]

**Appendix S2**: A descriptive summary of the variables from the baseline survey

| Survey Items | Total (n=100)† | |
| --- | --- | --- |
| *Sociodemographic Characteristics* |  | |
| Gender |  | |
| Male | 16/100 (16%) | |
| Female | 84/100 (84%) | |
| Ethnicity |  | |
| Han Chinese | 100/100(100%) | |
| Age |  | |
| 45 or below | 52/96^⊗^ (54%) | |
| Over 45 | 44/96^⊗^ (46%) | |
| Marital Status |  | |
| Married | 90/100 (90%) | |
| Divorced, unmarried and other | 10/100 (10%) | |
| Relationship with Adolescent Participating in Pilot |  | |
| Parent | 94/100 (94%) | |
| Grandparent, extended family and other | 6/100 (6%) | |
| Education Level |  | |
| Primary or below | 3/100 (3%) | |
| Secondary | 27/100 (27%) | |
| University and below | 70/100 (70%) | |
| Employment Status |  | |
| Employed | 85/100 (85%) | |
| Unemployed | 15/100 (15%) | |
| Annual Family Income |  | |
| 0-80000 RMB (<12560 USD) | 42/100 (42%) | |
| 80000-300000 RMB (12560-47096 USD) | 49/100 (49%) | |
| More than 300000 RMB (≥47096 USD) | 9/100 (9%) | |
| *Perceived Vaccination Benefits* |  | |
| “There is known history of HPV infection in my family” |  | |
| Yes | 3/100 (3%) | |
| No | 94/100 (94%) | |
| Unknown | 3/100 (3%) | |
| “There is known history of cervical cancer in my family” |  | |
| Yes | 1/100 (1%) | |
| No | 96/100 (96%) | |
| Unknown | 3/100 (3%) | |
| “I want to vaccinate my child to protect her against cervical cancer” |  | |
| Yes | 88/100 (88%) | |
| No | 12/100 (12%) | |
| “I believe HPV vaccine is important” |  | |
| Agree | 99/100 (99%) | |
| Disagree | 1/100 (1%) | |
| “I believe the vaccine is safe” |  | |
| Agree | 100/100(100%) | |
| Disagree |  | |
| “I believe the vaccine is effective” |  | |
| Agree | 99/99^∅^ (100%) | |
| Disagree |  | |
| *Perceived Vaccination Barriers* |  |  |
| “I have heard of negative news related to HPV vaccines in the media” |  | |
| Yes | 16/99^∅^ (16%) | |
| No | 83/99^∅^ (84%) | |
| “I have friends and/or family who oppose to getting the HPV vaccine” |  | |
| Yes | 12/99^∅^ (12%) | |
| No | 87/99^∅^ (88%) | |
| “People in my social circle have had bad experience with the HPV vaccine” |  | |
| Yes | 3/99^∅^ (3%) | |
| No | 96/99^∅^ (97%) | |
| “Cost of the vaccine is a barrier” |  | |
| Yes | 16/99^∅^ (16%) | |
| No | 83/99^∅^ (84%) | |
| “If the 9vHPV vaccine stock is insufficient at your community health centre, what would you do for your daughter” |  | |
| I would not get vaccinated now, and wait for resumption of 9vHPV vaccine stock then vaccinate | 74/100 (74%) | |
| I would vaccinate with 2vHPV or 4vHPV vaccines or use alternative methods to source vaccines | 26/100 (26%) | |
| “I would consider delaying vaccination for reasons other than allergy or ineligibility” |  | |
| Yes | 14/99^∅^ (14%) | |
| No | 86/99^∅^ (87%) | |
| *Sources of Health Information* |  | |
| “I have been given recommendations from friends and/or family” |  | |
| Yes | 29/100 (29%) | |
| No | 71/100 (71%) | |
| “I have been given recommendations from healthcare worker(s)” |  | |
| Yes | 26/100 (26%) | |
| No | 74/100 (74%) | |

⊗ *Missing data from four participants*

∅ *Missing data from one participant*

† *Data summarized as total frequencies due to both intervention and control arms of pilot study being comparable across all survey variables*
