# Supplementary material for: Between now and later: a mixed methods study of HPV vaccination delay among Chinese caregivers in urban Chengdu, China
Source: BMC Public Health. 2024 Jan 15;24:183. doi: 10.1186/s12889-024-17697-6 (PMC10790461; doi:10.1186/s12889-024-17697-6)
Supplement: Supplementary file 3 — Additional file 3: Appendix S3. Topic guide for focus group discussions. [file 12889_2024_17697_MOESM3_ESM.docx]

**Appendix S3**: Topic guide for focus group discussions

| Topic Area | Guiding and Probing Questions |
| --- | --- |
| Introduction | **What do you know about the HPV vaccine?**  - What is the primary function of the vaccine? Aside from cervical cancer, what are other benefits of the vaccine?  - What is your understanding of different “valents”?  - Have you heard of high-risk HPV before? |
| Perceived Susceptibility and Severity | **How likely do you think the HPV vaccine can prevent your daughter from getting infected with HPV? How about cervical cancer?**  - Is there are difference in effectiveness between 9v and 2vHPV? |
| Obtaining HPV information | **When and how did you become aware of the HPV vaccine?**  **Who do you trust the most for information about vaccines?**  **Did any events reported by the media/or news circulating within your social circle affect your decision to vaccinate your daughter, and if so, how?** |
| Perceived Benefits | **There are many different types of HPV vaccines in the market, do you think one vaccine is better than another?**  - Do you consider whether the vaccine is produced domestically or imported?  **Some people think it is better to vaccinate as early as possible, what do you think?**  **Some people believe the higher the “valent”/coverage the better the vaccine, what do you think?** |
| Adolescent Sexuality and Self-efficacy | **Experts recommend children should be vaccinated before sexual debut, how confident are you about making this decision for your daughter, that is to vaccinate before the sexual debut?** |
| Health Service Usage | **Does previous experience using health services (i.e., gynaecological check-ups) affect your decision to vaccinate your child?** |
| Cues to Action | **When making important health decisions for your child, do you consider the opinion of family and friends?**  **When making important health decisions for your daughter, do you consider the opinion of your partner?**  - What role did your partner play in this decision-making process?  **What do you think is the role of the health-care provider in parents’ decision to vaccinate their child? What advice was given to you?** |
| Structural Barriers and Perceived Barriers | **What are the significant barriers to getting your child vaccinated?** |
