# Supplementary material for: Between now and later: a mixed methods study of HPV vaccination delay among Chinese caregivers in urban Chengdu, China
Source: BMC Public Health. 2024 Jan 15;24:183. doi: 10.1186/s12889-024-17697-6 (PMC10790461; doi:10.1186/s12889-024-17697-6)
Supplement: Supplementary file 4 — Additional file 4: Appendix S4. Good Reporting of A Mixed Methods Study (GRAMMS) Checklist [40]. [file 12889_2024_17697_MOESM4_ESM.docx]

**Appendix S4:** Good Reporting of A Mixed Methods Study (GRAMMS) Checklist (40)

| **Guideline** |
| --- |
| Justification to use a mixed methods approach to the research question |
| Articulation of the design in terms of purpose, priority, and sequence of methods |
| Describe each method in terms of sampling, data collection and analysis |
| Delineate where and how integration occurs and who has participated in it |
| Describe any limitation of one method associated with the presence of another |
| Describe insights gained from mixing or integrating methods |
