# Supplementary material for: Between now and later: a mixed methods study of HPV vaccination delay among Chinese caregivers in urban Chengdu, China
Source: BMC Public Health. 2024 Jan 15;24:183. doi: 10.1186/s12889-024-17697-6 (PMC10790461; doi:10.1186/s12889-024-17697-6)
Supplement: Supplementary file 5 — Additional file 5: Appendix S5. A summary of the quotes from the qualitative analysis and their sources. [file 12889_2024_17697_MOESM5_ESM.docx]

**Appendix S5:** A summary of the quotes from the qualitative analysis and their sources

| Themes / Subthemes | Example Quotations and Source |
| --- | --- |
| Perceived benefits of vaccination | “I know that the vaccine can prevent against cancer. Cervical malignancy really is terrifying and difficult to treat. This vaccine can protect women and our girls, and all we can do is protect ourselves.” *(Mother of vaccinated daughter, focus group 2)* |
| Preference for 9vHPV Vaccines | “Rather than fighting off two or four enemies, the 9vHPV vaccine is capable of fighting nine enemies in one go.” (*Mother of vaccinated daughter, focus group 1*) |
|  | “If we were unable to get the 9vHPV vaccine at schools, which is the best available option, we can only make do with a lesser version, like getting the 2vHPV option.” *(Mother of vaccinated daughter, focus group 1)* |
|  | “When I attended the appointment, I had no idea what to choose for my daughter. I was just going to get whatever other people got because I had no idea.” *(Mother of vaccinated daughter, focus group 1)* |
|  | “This is for the good of my child. I am willing to pay a higher cost as long as I can get the best vaccine for her.”(*Mother of unvaccinated daughter, focus group 3)* |
|  | “As parents, we know that the [9vHPV] vaccine is the best option available for children, so I will choose it. I don’t agree with getting multiple [vaccination] regimes because it’s not good for the child’s development and growth.” *(Mother of vaccinated daughter, focus group 1)* |
|  | “We will do our best to get the best available option for our child. We heard reports that it is difficult to get this popular vaccine on the market, but that’s because good things are hard to get.” *(Mother of vaccinated child, focus group 1)* |
| Barriers: too much information | “There are now many channels to get information [about the HPV vaccines] compared to when we were young. There are many different kinds of media, which can be overwhelming and complex.” (*Mother of unvaccinated daughter, focus group 3)* |
|  | “Most of the information is passed on by word of mouth because it is a hot topic among our friends. It’s hard to trust all the information – we must take everything that was said with a grain of salt.” *(Mother of vaccinated daughter, focus group 1)* |
| Barriers: vaccine shortage | “Everyone, from the government to the health professionals, all said the 9vHPV vaccine was the best. We were told to get vaccinated as early as possible, but this was impossible because there were no vaccines available!” (*Mother of vaccinated daughter, focus group 1)* |
| Barriers: vaccine safety | “I think most of us parents don’t care whether the vaccine is imported or domestically manufactured. What we care about is vaccine safety. There is no guarantee of safety for imported vaccines either.” *(Mother of vaccinated daughter, focus group 1)* |
| Barriers: perceived sexual inactivity of adolescent girls | “16 years old is an ideal age to get vaccinated because it satisfies the age eligibility criteria, and we can get this done before she goes to university. After kids leave for university, no matter how strict you are with them before, everything is out of your control. I cannot guarantee she will not engage in sexual activity then, can I?” *(Mother of vaccinated daughter, focus group 2)* |
|  | “Before the age of 18, children are at school or at home so everything is under control because they’re under our noses all the time.” *(Mother of unvaccinated daughter, focus group 3)* |
| Role of parents | “I tried everything I could to get an appointment for the 9vHPV vaccine. I failed, so I got my husband involved to help fight for appointment quotas … He didn’t even know the vaccine exists – never heard of it before – fathers don’t pay attention to this stuff. Anyway, I got him to help, and he got really frustrated because obviously he also failed, then he showed some interest and read up on it.” *(Mother of vaccinated daughter, focus group 2)* |
